# Comparisons of In Vivo and In Vitro Opioid Effects of Newly Synthesized 14-Methoxycodeine-6-*O*-sulfate and Codeine-6-*O*-sulfate

**DOI:** 10.3390/molecules25061370

**Published:** 2020-03-17

**Authors:** Ferenc Zádor, Amir Mohammadzadeh, Mihály Balogh, Zoltán S. Zádori, Kornél Király, Szilvia Barsi, Anna Rita Galambos, Szilvia B. László, Barbara Hutka, András Váradi, Sándor Hosztafi, Pál Riba, Sándor Benyhe, Susanna Fürst, Mahmoud Al-Khrasani

**Affiliations:** 1 Department of Pharmacology and Pharmacotherapy, Faculty of Medicine, Semmelweis University, Nagyvárad tér 4, P.O. Box 370, H-1445 Budapest, Hungary; zador.ferenc@pharma.semmelweis-univ.hu (F.Z.); mohammadzadeh.amir@med.semmelweis-univ.hu (A.M.); baloghmisi90@gmail.com (M.B.); zadori.zoltan@med.semmelweis-univ.hu (Z.S.Z.); kiraly.kornel@med.semmelweis-univ.hu (K.K.); szilvi.barsi@gmail.com (S.B.); galambos.anna@pharma.semmelweis-univ.hu (A.R.G.); laszlo.szilvia@med.semmelweis-univ.hu (S.B.L.); hutka.barbara@semmelweis-univ.hu (B.H.); riba.pal@med.semmelweis-univ.hu (P.R.); furst.zsuzsanna@med.semmelweis-univ.hu (S.F.); 2 Institute of Biochemistry, Biological Research Center of the Hungarian Academy of Sciences, Temesvári krt. 62., H-6726 Szeged, Hungary; benyhe.sandor@brc.hu; 3 Department of Pharmaceutical Chemistry, Semmelweis University, Hőgyes Endre u., 9. H-1092 Budapest, Hungary; uvamortifera@gmail.com (A.V.); hosztafi.sandor@pharma.semmelweis-univ.hu (S.H.)

**Keywords:** peripheral antinociception, 14-methoxycodeine-6-*O*-sulfate, codeine-6-*O*-sulfate

## Abstract

The present work represents the in vitro (potency, affinity, efficacy) and in vivo (antinociception, constipation) opioid pharmacology of the novel compound 14-methoxycodeine-6-*O*-sulfate (14-OMeC6SU), compared to the reference compounds codeine-6-*O*-sulfate (C6SU), codeine and morphine. Based on in vitro tests (mouse and rat vas deferens, receptor binding and [^35^S]GTPγS activation assays), 14-OMeC6SU has µ-opioid receptor-mediated activity, displaying higher affinity, potency and efficacy than the parent compounds. In rats, 14-OMeC6SU showed stronger antinociceptive effect in the tail-flick assay than codeine and was equipotent to morphine, whereas C6SU was less efficacious after subcutaneous (s.c.) administration. Following intracerebroventricular injection, 14-OMeC6SU was more potent than morphine. In the Complete Freund’s Adjuvant-induced inflammatory hyperalgesia, 14-OMeC6SU and C6SU in s.c. doses up to 6.1 and 13.2 µmol/kg, respectively, showed peripheral antihyperalgesic effect, because co-administered naloxone methiodide, a peripherally acting opioid receptor antagonist antagonized the measured antihyperalgesia. In addition, s.c. C6SU showed less pronounced inhibitory effect on the gastrointestinal transit than 14-OMeC6SU, codeine and morphine. This study provides first evidence that 14-OMeC6SU is more effective than codeine or C6SU in vitro and in vivo. Furthermore, despite C6SU peripheral antihyperalgesic effects with less gastrointestinal side effects the superiority of 14-OMeC6SU was obvious throughout the present study.

## 1. Introduction

Natural, semisynthetic and synthetic μ type opioid receptor (MOR) agonists are essential and the most efficient medicines to relieve moderate to severe pain in the clinical practice. MORs, together with δ and κ type opioid receptors (abbreviated as DOR and KOR, respectively), are G_i/o_-type G-protein coupled receptors, which reduce cAMP levels, inhibit calcium channels (N/P) and open potassium channels, overall resulting in the inhibition of neurotransmitter release from the presynaptic membrane [1,2] and hyperpolarizing the post synaptic membrane [3]. From a general point of view, opioids produce analgesia by full or partial activation of opioid receptors, in particular MOR types at the spinal and supraspinal levels. In addition, pharmacological evidence has shown that opioids are also capable to produce antinociception by the activation of opioid receptors reside outside the central nervous system [4,5,6,7,8,9,10]. Indeed, opioid agonists that have been reported to induce peripheral antinociception display a prerequisite physiochemical property, a limited central nervous system (CNS) penetration [4,5,7]. Thus, over the last four decades many opioid research groups have undertaken the task of synthesis and pharmacological characterization of opioid compounds with limited CNS penetration and thereby inducing antinociception by activating opioid receptors at the periphery. It is worth noting that quaternization of nitrogen on morphine-based structure derivatives has been reported to have negative impact on both the affinity and agonist activity of generated analogs [11]. To overcome this issue, medicinal chemists have been seeking for other ways to develop opioid agonists with limited CNS penetration, meanwhile retaining or even increasing the pharmacological profile, when compared to the parent molecules [12].

One of the most successful chemical approaches to improve the safety (limited CNS penetration) profile and the efficacy of morphine and other morphinans, is the synthesis of zwitterionic structures. This chemical structure modification was carried out by the introduction of ionizable groups into the C-6 position such as sulfate, amino acids or guanido groups as described previously [6,13,14,15,16,17,18,19,20,21]. Such compounds have been demonstrated to have reduced central side effects as well [5,21]. Extreme increase in the affinity, efficacy and analgesic activity was measured for 14-methoxy analogs of morphine or oxymorphone compared to the parent compounds [4,6,20,22,23]. 

A study by Zuckerman and co-workers has shown that codeine-6-*O*-sulfate (C6SU) displayed exclusive affinity for MOR, yet it was found that intracerebroventricularly administered C6SU produced weak analgesia at lower doses and increasing the dose of C6SU evoked convulsion and death that hampered the assessment of its analgesia [17]. Indeed, convulsion following intracerebroventricular (i.c.v.) injection of codeine has also been reported, though systemic codeine is used as an analgesic for mild to moderate pain and the World Health Organization approves its usage as the second step of the analgesic ladder for cancer pain [24]. To the best of our knowledge, no data have been reported on the systemic analgesic effect of C6SU.

Compared to morphine, codeine has a weaker affinity for opioid receptors and preferably binds to the MOR [25,26,27,28]. As with all opioids, codeine also produces central adverse effects such as addiction and respiratory depression [26,29,30,31], as well as peripheral ones such as constipation [30,31]. These are mainly mediated through the MOR [32,33]. It is therefore imperative to increase the analgesic efficacy of C6SU by the above mentioned strategy and underline again the analgesic properties of C6SU and codeine after both central and peripheral administrations.

**Figure 1:**
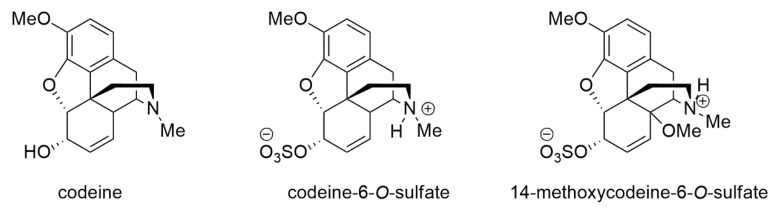
The structure of codeine, codeine-6-*O*-sulfate and 14-methoxycodeine-6-*O*-sulfate.

The aims of the present work were to synthesize and characterize 14-methoxycodeine-6-*O*-sulfate (14-OMeC6SU) and to compare the structure-activity relationship of the novel compound to the already characterized C6SU [14,17], to the parent compound codeine (for structures see Figure 1) and to morphine, a prototypical opioid analgesic. During pharmacological characterization we analyzed its receptor preference (selectivity and affinity) by biochemical (equilibrium radioligand competition binding) and by biological assays (MVD, mouse vas deferens; RVD, rat vas deferens). A further objective was to measure the analgesic effect of the novel compound and compare to C6SU, codeine and morphine in rat tail-flick test. We have also characterized the antinociceptive effects of 14-OMeC6SU and C6SU on hyperalgesia induced by Complete Freund’s Adjuvant (CFA) in order to draw a conclusion on the possible peripheral antinociception. Additionally, constipation, one of the most common side effects of codeine was also investigated, by analyzing the changes in gastrointestinal transit with the charcoal meal assay in the presence of the test compound.

## 2. Results

### 2.1. Receptor Binding Assays

#### 2.1.1. 14-OMeC6SU Displayed High Affinity and MOR Selectivity in Radioligand Competition Binding Assay

The novel codeine analog 14-OMeC6SU, CS6U and the parent compound codeine were tested for opioid receptor binding affinity and selectivity in in vitro radioligand competition binding assays using prototypic selective radioligands for MOR and DOR in rat brain membrane homogenates, or for KOR in guinea pig brain membrane homogenates. The prototypic opioid ligands displayed high affinity as expected and are in accordance with previous data [20,23].

**Figure 2:**
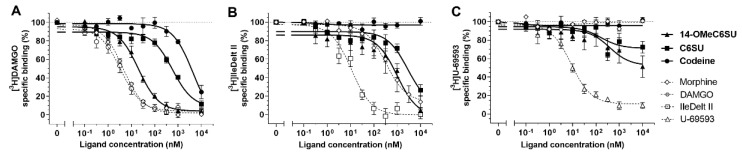
Dose-response curves featuring binding affinity of 14-methoxycodeine-6-*O*-sulfate (14-OMeC6SU) and codeine-6-*O*-sulfate (C6SU) to µ-opioid receptor (**A**), δ-opioid receptor (**B**) and κ-opioid receptor (**C**) compared to codeine and morphine in competition binding experiments performed in rat (**A** and **B**) and guinea pig (**C**) brain membrane homogenate. For control the unlabeled form of the applied radioligands are also indicated. All figures represent the specific binding of the corresponding radioligand (**A**: [^3^H]DAMGO, **B:** [^3^H]Ile^5,6-^deltorphin II [IleDelt II], **C:** [^3^H]U-69593) in percentage (means ± S.E.M.) normalized to total specific binding (100%) in the presence of increasing concentrations (0.1 nM–10 μM) of the indicated unlabeled ligands. Total specific binding was determined in the absence of the indicated unlabeled ligands and indicated with a dotted line. The K_i_ ± S.E.M. values are presented in Table 1.

14-OMeC6SU showed higher affinity for MOR 28 and 217 times when compared to C6SU and codeine, respectively (Figure 2A, Table 1). In addition, it displayed affinity for DOR and KOR but the affinity was 101 and 72 fold, respectively, lower than that for MOR. (Figure 2B, Table 1). Although 14-OMeC6SU displayed relevant KOR affinity, the displacement was only 51.34% (±11.53) compared to total specific binding (Figure 2C, Table 1). Compared to codeine, C6SU showed significantly higher MOR affinity and endowed a micromolar affinity to the DOR, however, C6SU did not displace the DOR radioligand completely to the non-specific binding level at the highest concentration (26.45 ± 6.02%, Figure 2B). Furthermore, in contrast to 14-OMeC6SU, C6SU did not show significant KOR affinity (Figure 2C, Table 1). The parent compound codeine showed poor affinity for MOR and none for DOR and KOR (Figure 2 and Table 1). 

#### 2.1.2. 14-OMeC6SU Shows Strong Agonist Activity in [^35^S]GTPγS Binding Assay

**Figure 3:**
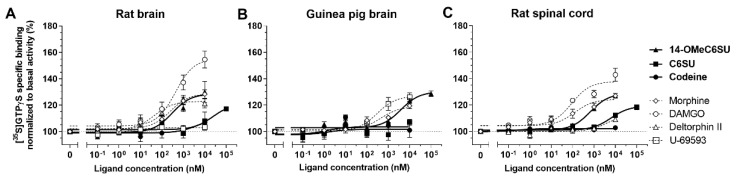
14-methoxycodeine-6-*O*-sulfate (14-OMeC6SU) compared to codeine-6-*O*-sulfate (C6SU) and codeine in [^35^S]GTPγS binding assays performed in rat (**A**) and guinea pig (**B**) brain and rat spinal cord (**C**) membrane homogenates. For comparison standard µ-, δ- and κ-opioid receptor selective agonists, DAMGO, deltorphin II (Delt II) and U-69593, respectively are also presented. Figures represent the specific binding of [^35^S]GTPγS in percentage (means ± S.E.M.) in the presence of increasing concentrations (0.1 nM–100 µM) of the indicated ligands. “Total” on the x-axis indicates the basal activity of the monitored G-protein (defined as 100%, its level is presented as a dotted line), which is measured in the absence of the compounds and also represents the total specific binding of [^35^S]GTPγS. E_max_ and EC_50_ ± S.E.M. values are presented in Table 2.

The agonist activity of 14-OMeC6SU was analyzed in [^35^S]GTPγS G-protein activity assay and compared to C6SU, codeine and the prototypical opioid receptor selective agonists for µ, δ and κ opioid receptors (DAMGO, deltorphin II and U-69593, respectively) (Figure 3 and Table 2). Similar to competition binding experiments, rat and guinea pig brain tissues were used to measure the agonist potency and efficacy of the test compounds. In addition, 14-OMeC6SU was also measured in rat spinal cord and compared to MOR and DOR selective agonists. The agonist properties (EC_50_, E_max_,) of the reference compounds were as expected and as reported previously [20,23]. Additionally, in rat brain tissues we aimed to demonstrate whether U-69593 can produce measurable KOR activity. In accordance with previous work [34] the KOR agonist did not show significant agonist activity (Figure 3A, Table 2) in contrast to guinea pig brain (Figure 3B, Table 2).

14-OMeC6SU and DAMGO showed comparable agonist potencies, but 14-OMeC6SU displayed lower E_max_ value than DAMGO (Figure 3, Table 2). On the other hand, 14-OMeC6SU displayed similar agonist efficacy (E_max_) in rat and guinea pig brain or rat spinal cord tissues, but the potency of the compound was significantly weaker in guinea pig brain membranes (Figure 3, Table 2). C6SU showed partial agonist activity in rat brain or spinal cord and failed to produce agonist effect in guinea pig brain (Figure 3, Table 2). Codeine did not alter G-protein basal activity, thus it did not show agonist activity in any of the investigated samples (Figure 3, Table 2). 

14-OMeC6SU showed naloxone reversible effect in rat brain or guinea pig brain, indicating that the test compound produces its effect through the opioid receptors (Table 3). 

### 2.2. 14-OMeC6SU Is a Full Agonist in MVD and RVD

**Figure 4:**
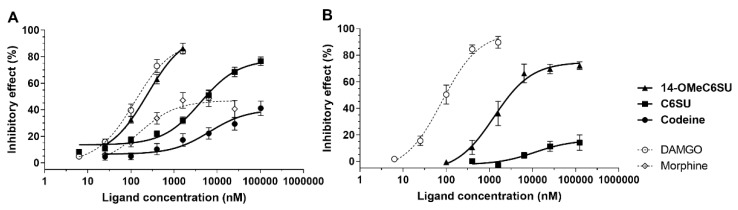
The inhibitory effect of 14-OMeC6SU on electrically evoked contractions of MVD (panel **A**) or RVD (panel **B**) compared to C6SU, codeine, morphine or DAMGO. Data are presented as mean ± S.E.M. The E_max_ and EC_50_ values are presented in Table 4.

14-OMeC6SU in a concentration dependent manner, inhibited the mouse vas deferens smooth muscle contractions (Figure 4A). The measured E_max_ (efficacy) was similar to that of DAMGO, however DAMGO was 2 times more potent than 14-OMeC6SU (Table 4, Figure 4A). 14-OMeC6SU showed significant efficacy compared to C6SU, codeine or morphine in inhibition of the contraction of MVD (Table 4, Figure 4A). C6SU, similar to morphine, showed concentration-response curves reaching a ceiling effect in a submaximal range (Table 4, Figure 4A). The EC_50_ of test compounds are presented in Table 4.

The opioid receptor type preference of 14-OMeC6SU was assessed in the MVD assay in the presence of naloxone as non-selective opioid antagonist. Furthermore, 14-OMeC6SU receptor preference was also examined in the presence of naltrindole or nor-BNI, selective antagonist for DOR or KOR, respectively. For comparison, the prototype agonists, DAMGO, DPDPE and U-69593 for MOR, DOR and KOR, respectively were also used. The K_e_ values of the antagonists are presented in Table 5. The obtained K_e_ values of naloxone against 14-OMeC6SU, C6SU or DAMGO were not significantly different from one another, indicating that the test compounds act on MOR. 

In RVD, 14-OMeC6SU produced 74.81 ± 2.74% maximum effect (efficacy), which is significantly less than in MVD bioassay (98.31 ± 0.52%). Statistical analysis revealed that the efficacy of the novel compound significantly decreased compared to DAMGO (E_max_: 97.57 ± 4.52%), though the fall in the values did not exceed 25% indicating that the novel compound produced substantial efficacy in this organ. On the other hand, the E_max_ of C6SU did not exceed 20%, showing that there was a pronounced drop in the efficacy compared to the novel compound or DAMGO (Table 4, Figure 3B). Morphine and codeine failed to produce any inhibitory effect in RVD. The K_e_ values of naloxone against 14-OMeC6SU and DAMGO did not differ significantly from each other (Table 5), indicating a MOR-mediated effect. 

### 2.3. 14-OMeC6SU Produces Antinociceptive Effect in Rat Tail-flick Assay 

**Figure 5:**
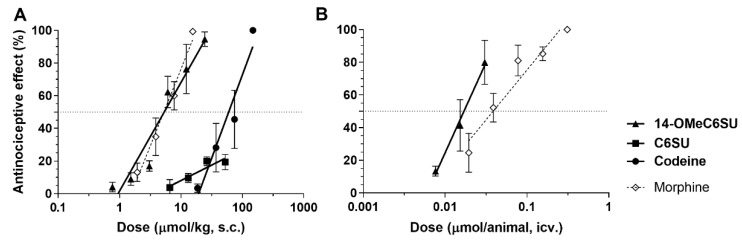
The antinociceptive effect (see Table 3 and Table 4) of 14-OMeC6SU, compared to C6SU codeine and morphine in rat tail-flick test after s.c. (**A**) and i.c.v. (**B**) administration. Data represent means ± S.E.M.

Following the in vitro characterization, rat tail-flick test as an in vivo thermal pain model was applied to examine the acute antinociceptive effect of 14-OMeC6SU, and to compare it that of C6SU, codeine or morphine. After s.c. administration 14-OMeC6SU showed antinociceptive effect equipotent to morphine and stronger than codeine (Table 6, Figure 5A). The test compounds achieved peak effect at 30 min. C6SU showed weak antinociception displaying a ceiling effect, which did not reach 20% (19.91 ± 2.67 at 26.35 µmol/kg; Figure 5A). ED_50_ values (µmol/kg) of 14-OMeC6SU, codeine and morphine were 5.33, 54.01 and 6.87, respectively (Table 6). Additionally, the antinociceptive effect of 12.21 µmol/kg 14-OMeC6SU (76.42% ± 9.08; n = 10) was completely blocked by co-administered 3.06 µmol/kg naloxone (0.01% ± 5.91; n = 5). 

After i.c.v. administration the peak effects of 14-OMeC6SU and morphine were achieved at 10 and 30 min, respectively (Table 7, Figure 5B). Of note, in accordance with previous studies [17], i.c.v. C6SU caused convulsions which hampered the assessment of its antinociception. The ED_50_ values (µmol/animal) of 14-OMeC6SU and morphine were 0.017 and 0.039, respectively (Table 7).

### 2.4. 14-OMeC6SU (in Certain Doses) and C6SU Possess Peripheral Antinociceptive Effects after Systemic Administration in Rats with CFA-Induced Inflammatory Pain

**Figure 6:**
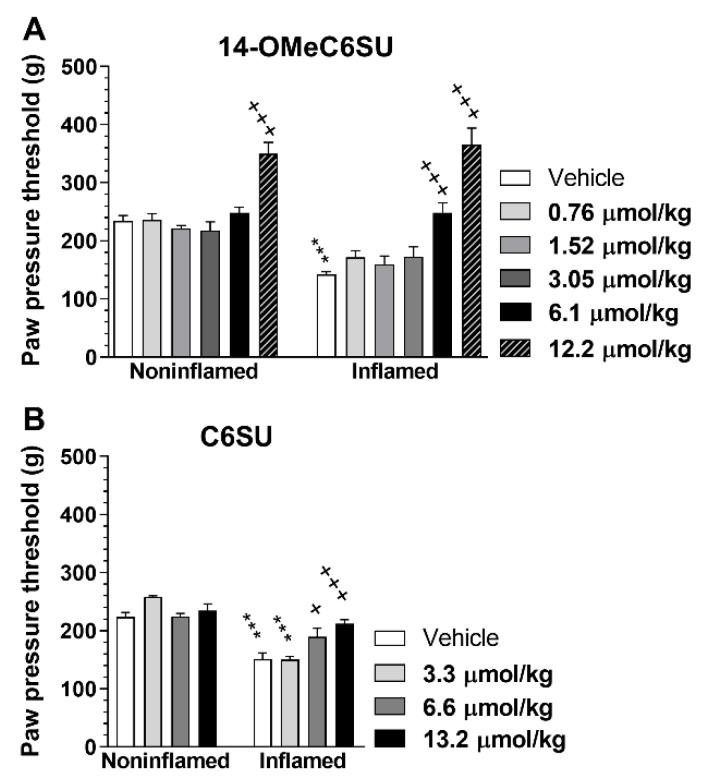
The peak antinociceptive effects of subcutaneously administered 14-OMeC6SU (**A**) and C6SU (**B**) in Complete Freund’s Adjuvant (CFA)-induced inflammatory pain. Figures indicate the paw pressure threshold in g-s in the presence of 14-OMeC6SU or C6SU in the indicated dosages 30 min after the injection of the tests compounds. Data represent means ± S.E.M. (n = 5–11 per group). * significant difference between inflamed and noninflamed paw within the corresponding treated group (two-way ANOVA, Sidak’s multiple comparisons test). ^+^ significant difference compared to inflamed paw of vehicle treated group or compared to vehicle and 14-OMeC6SU 6.1 µmol/kg in the noninflamed (two-way ANOVA, Tukey’s multiple comparisons test). ***^/+++^ *P* < 0.001; ^+^ *P* < 0.05.

**Figure 7:**
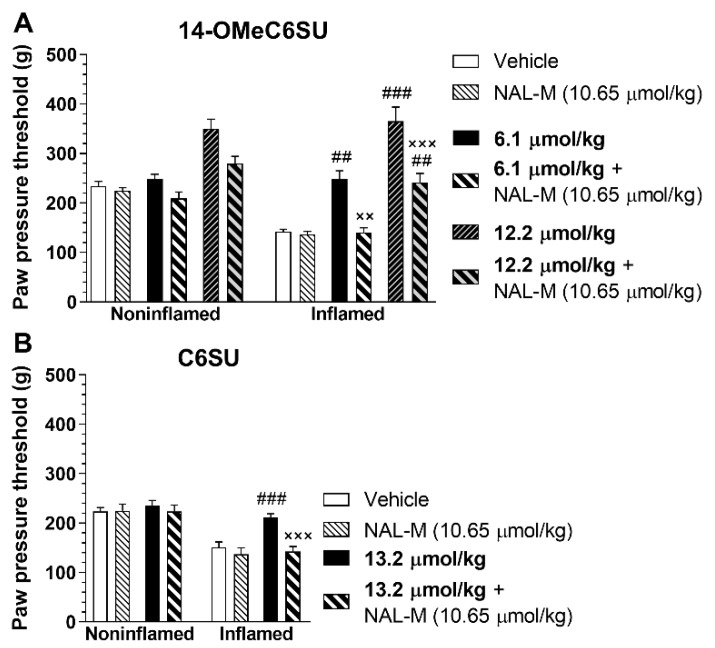
Antagonist action of co-administered naloxone methiodide (NAL-M) on the antinociceptive effect of 14-OMeC6SU (**A**) and C6SU (**B**) in CFA-induced inflammatory pain 30 min after the injection of the compounds. Figures indicate the paw pressure threshold in g-s in the presence of 14-OMeC6SU, C6SU and NAL-M, and in the presence of NAL-M co-administered with 14-OMeC6SU or C6SU in the indicated dosages. Data represent means ± S.E.M. (n = 5–11 per group). ^#^ significant difference within the inflamed paw compared to vehicle, NAL-M and to 6.1 µmol/kg 14-OMeC6SU + NAL-M in panel A (two-way ANOVA, Tukey’s multiple comparisons test). ^×^ significant difference within the inflamed paw compared to the correspondent test compound alone in the appropriate dose (two-way ANOVA, Tukey’s multiple comparisons test). ^××/##^ *P* < 0.01; ^×××/###^ *P* < 0.001.

The antinociceptive effect of 14-OMeC6SU was further investigated in CFA-induced inflammatory pain, using Randall-Selitto paw pressure test. The paw pressure threshold (PPT) was reduced in the inflamed right paw by 54.38 ± 1.75 (n = 21) after the 4th day and by 61.59 ± 2.11 (n = 21) after 7th day following CFA treatment. The antinociceptive effect of 14-OMeC6SU was measured in doses of 0.76, 1.52, 3.05, 6.1 and 12.2 µmol/kg, 30 and 60 min after s.c. treatment both in inflamed and noninflamed paws (Figure 6A). 14-OMeC6SU in dose of 6.1 µmol/kg abolished hyperalgesia in the inflamed paws and having no effect on the noninflamed paws, indicating that the effect was localized to the inflamed one (Figure 6A). Furthermore, the antihyperalgesic effect of this dose was abolished by co-administered naloxone methiodide (NAL-M; 10.65 µmol/kg), the peripheral restricted opioid antagonist (Figure 7A). In the applied dose NAL-M failed to affect the pain thresholds of either the inflamed or noninflamed paws (Figure 7A). Higher dose of 14-OMeC6SU (12.2 µmol/kg) produced significant increase in the pain threshold of both inflamed and noninflamed paws after 30 min (Figure 6A), and this effect was partially affected by NAL-M in the inflamed paw (Figure 7A). Since C6SU produced weak antinociception but free of CNS effects such as convulsion in rat tail-flick test after s.c. administration, it was also further investigated in a similar setup. The test doses of s.c. C6SU were 3.3, 6.6 and 13.2 µmol/kg. Similar to 14-OMeC6SU, C6SU reached antinociceptive peak effect after 30 minutes. C6SU in doses of 6.6 and 13.2 µmol/kg produced significant antihyperalgesic action in the inflamed paw and failed to affect the noninflamed paws (Figure 6B). In contrast to 14-OMeC6SU, C6SU at higher doses produced an effect only in the inflamed paw. In addition, the impact of the highest dose of C6SU was reversed by 10.65 µmol/kg NAL-M (Figure 7B). Vehicle failed to affect either the inflamed or noninflamed paws.

### 2.5. Inhibitory Effect of Systemic 14-OMeC6SU on Gastrointestinal Transit in Rats

**Figure 8:**
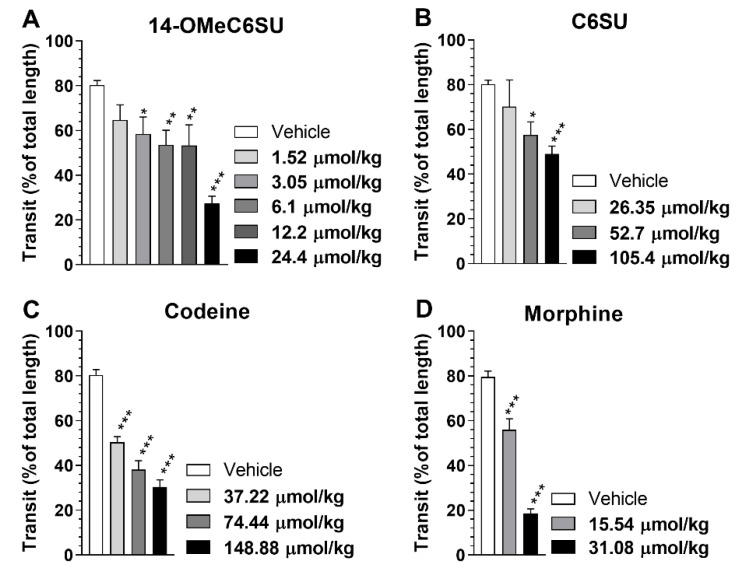
The effect of 14-OMeC6SU (**A**) on rat gastrointestinal transit compared to C6SU (**B**), codeine (**C**) and morphine (**D**). The figure represents the inhibition of gastrointestinal transit in percentage (means ± S.E.M.) of total length compared to vehicle treated group in the presence of 14-OMeC6SU, C6SU and morphine in the indicated dosages. * indicates the significant difference compared to control (One-way ANOVA, Tukey’s multiple comparisons post hoc test, *P* < 0.001). * *P* < 0.05; ** *P* < 0.01; *** *P* < 0.001.

After s.c. administration, 14-OMeC6SU up to 12.2 µmol/kg induced mild, but statistically significant inhibition of the gastrointestinal transit, whereas at higher dose (24.4 µmol/kg) it evoked a marked (~68%) inhibition compared to vehicle (Figure 8A). C6SU displayed significant inhibitory effect only in higher, 52.7 µmol/kg, which effect was comparable to that of 14-OMeC6SU and morphine in 12.2 and 15.54 µmol/kg dose, respectively (Figure 8B). C6SU at a higher, 105.4 µmol/kg dose further inhibited gastrointestinal transit, although the effect was not as strong as seen with 14-OMeC6SU with the highest applied dose (Figure 8A and B). Codeine, similar to C6SU was examined in higher doses, however it showed a more pronounced effect, than C6SU (Figure 8C and B). On the other hand, 148.88 µmol/kg codeine showed a similar level of inhibitory effect with 24.4 µmol/kg 14-OMeC6SU, which is more than a 6-fold difference in the doses (Figure 8C and A). As expected, 31.08 µmol/kg morphine induced pronounced inhibition of the gastrointestinal transit (Figure 8D). 

## 3. Discussion

In this study for the first time we present the in vitro and the in vivo pharmacological properties of 14-OMeC6SU applying biochemical, biological (isolated organs), acute and sub chronic pain model assays. We also studied the acute effect of 14-OMeC6SU compared to C6SU or the clinically established analgesic compound, morphine on rat intestinal transit. The in vitro results (affinity, potency and efficacy) are alongside with the superiority of the novel compound, 14-OMeC6SU over C6SU or codeine. Interestingly, results of in vivo studies for C6SU, particularly which obtained in inflammatory pain model or in gastrointestinal transit assay, are of potential benefit in clinical practice: peripheral analgesia and lower gastrointestinal adverse effect compared to 14-OMeC6SU. However, in terms of analgesic potency 14-OMeC6SU was more potent than the C6SU in the applied pain models (thermal and inflammatory pain) under the present experimental circumstances.

*In vitro* assays were applied to assess the affinity, agonist potency, efficacy and receptor preference (selectivity) of 14-OMeC6SU compared to that of parent or reference compounds. The consequence of the chemical modification, the introduction of methoxy group into the C-14 of C6SU resulted in a significant increase in the efficacy. Taking a backward step, the chemical modification carried on codeine by Zuckerman [17], namely the introduction of -OSO_3_ into C-6 of codeine has improved both the affinity and the potency, yet limited the CNS access following systemic administration. Nonetheless, both compounds, C6SU and codeine showed very low affinity for DOR (C6SU) and had no measurable affinity for KOR. On the other hand, 14-OMeC6SU beside its affinity for MOR displayed also measurable affinity for DOR and KOR. These results are reflected by the dose ratios produced by naloxone, naltrindole and nor-BNI against 14-OMeC6SU, which were 9, 4 and 5, respectively. Accordingly, 14-OMeC6SU showed the highest affinity for MOR compared to C6SU or codeine and displayed similar binding properties as described in earlier studies, together with codeine [14,17,27]. Our results correspond well with previous data and confirm that the 14-*O*-methylation of the morphinan structure significantly enhances not only the affinity for MOR, but also for DOR and KOR [4,6,20,22,23]. Other research groups have reported that 14-*O*-methyl analogs of oxymorphone have improved affinity, agonist activity and antinociceptive potency compared to their parent compound, oxymorphone [36]. Indeed, a positive tremendous impact on the affinity and efficacy of morphine has been achieved following introduction of -OSO_3_ and *O*-methyl into C-6 and C-14, respectively [22]. 

The analgesic efficacy of opioid agonists against acute and subchronic inflammatory pain has been established in broad panel of human studies and rodent pain models. However, the achieved analgesia following systemic administration of currently available opioids in clinical practice is a CNS-mediated action, though a large number of studies carried out on humans and rodents has demonstrated the presence of functionally active peripheral opioid receptors [6,16,37,38]. As a result, substantial research has been undertaken to synthesize a new generation of peripheral opioid receptor agonists free of central adverse effects (respiratory depression, addiction, tolerance, etc.), since the opioid overdose related deaths are stemmed from such mechanisms, particularly respiratory depression. Therefore, it is an unmet medical need that require new inventive tools to solve the current opioid overdose crisis. It can be speculated that opioid ligands with limited CNS penetration may produce analgesia through the activation of opioid receptors reside outside the CNS, namely on sensory neurons at the periphery. Previously, Schmidhammer and Spetea and their coworkers have developed several 14-alkyloxymorphinan and 14-*O*-methyloxymorphone analogs to improve the pharmacology and safety profile of such compounds [36,39]. A similar strategy was carried out for the synthesis of 14-*O*-methylmorphin-6-*O*-sulfate by our group [22] and here for the novel codeine analog, 14-OMeC6SU. Herein, we found that 14-OMeC6SU, codeine and morphine produced dose-dependent antinociception in acute thermal pain model. On the other hand, C6SU showed antinociceptive dose-response curve of ceiling effect (maximum effect was 20%). We can hypothesize that this is due to both the pharmacodynamic and pharmacokinetic properties of C6SU. The former feature reflects the efficacy of C6SU, meaning that it activates the peripheral opioid receptors which can produce only submaximal analgesia. The observed weak antinociceptive response for C6SU is supported by data obtained from experiments carried out in [^35^S]GTPγS binding assays as well as in the MVD or RVD. For example, in RVD C6SU showed E_max_ (efficacy) that could not exceed 20 percent of response achieved by DAMGO, a highly selective MOR agonist with high efficacy demonstrated in in vitro and in vivo animal experiments [37,40,41].

The latter feature (pharmacokinetic) indicates that the possible access of C6SU into the brain is limited, because the injection of C6SU directly into the brain evoked convulsion observed in our present experiments and also reported by Zuckerman et al. [17]. At the present, we speculate that in the rat tail-flick test, which is an acute thermal pain model and MORs are not undertaken to substantial changes related to their number, the peripheral MOR reserve is not large enough for C6SU to produce stronger analgesic effect. Therefore, based on the above, we further extended our studies to examine the effect of 14-OMeC6SU compared to C6SU in animal pain model whereas the MORs reserve is significantly up-regulated [7,42,43]. Thus, CFA-induced inflammatory pain model suits the prerequisite condition for partial agonists—like C6SU in the present work—because ours and other research groups have reported on increased peripheral MOR expression in the inflamed paw in this model [37]. It is well known, that partial agonists show lower agonist activity than the higher efficacy opioid agonists when the MOR reserve is decreased [40]. Additionally, this pain model is widely used in opioid research to assess the contribution of the peripheral opioid receptors to antinociception [5,44]. Here we found that systemic administration of both 14-OMeC6SU and C6SU abolished the reduced pain threshold of the inflamed paws, indicating the antihyperalgesic effects of the test compounds. Of note, the antihyperalgesic effect of 14-OMeC6SU which is limited to inflamed paw was achieved by two times smaller dose than that achieved by C6SU. This result indicates that the introduction of 14-methoxy in C6SU enhanced the antinociceptive effect in accordance with our and results obtained by Schmidhammer and co-workers [36]. To examine whether this effect is peripherally related, we applied systemic naloxone methiodide, a peripherally acting opioid antagonist [45,46,47]. Accordingly, 14-OMeC6SU only in 6.1 µmol/kg dose produced naloxone methiodide reversible antihyperalgesic effect. However, co-administered NAL-M failed to antagonize the antihyperalgesic effect of 12.2 µmol/kg 14-OMeC6SU, indicating the involvement of MORs within the CNS. We have paid attention to the chosen dose of NAL-M when we designed the experiments. The 10.65 µmol/kg NAL-M dose was chosen based on results published by Fürst and Schmidhammer groups as well as by Lackó et al. [5,6]. In the work by Fürst et al., the applied NAL-M dose was five times less than we used here and ten times less than used by Lackó et al. [5]. Keeping in mind that the HS-731, a MOR agonist of high efficacy with limited CNS penetration, was proved to be 209 times more potent analgesic agent than morphine after s.c. administration. In the present study applying a similar animal pain model, 14-OMeC6SU produced equivalent analgesia with morphine following s.c. administration. NAL-M at five times lower dose than in the present study as mentioned above, was able to antagonized the effect of the highly potent opioid agonist, HS-731. It therefore is unlikely that the applied NAL-M dose in our study was unable to fully antagonize the higher dose (12.2 µmol/kg) of 14-OMeC6SU, rather than the penetration of 14-OMeC6SU to the CNS [6]. Herein, C6SU in doses of 6.6 µmol/kg and 13.2 µmol/kg reversed the developed hyperalgesia in the inflamed paws and showed no significant impact on the noninflamed paws. Moreover, this effect was sensitive to the co-administered naloxone methiodide, indicating the peripheral mediated effect. In addition, no convulsive effect was observed in agreement with data obtained in the tail-flick test and in contrast to the convulsive effect reported by Zuckerman following i.c.v injection [17]. Of note, C6SU in s.c. dose of 13.2 µmol/kg abolished the CFA-induced hyperalgesia, whereas 105.4 µmol/kg produced weak antinociception (see tail-flick assay). It means that the dose of C6SU that peripherally abolishes hyperalgesia is 8 fold less than the highest dose applied in thermal pain model.

Furthermore, the possible impact of 14-OMeC6SU and C6SU on gastrointestinal transit was also investigated, since the activation of gut opioid receptors is the crucial mechanism involved in the development of constipation [48]. Opioid-induced constipation is a main issue in discontinuing treatment with opioids, though antagonists for reversal of opioid-induced constipation such as methylnaltrexone have been approved [49]. Indeed, peripherally acting opioids of lower inhibitory effect on gastrointestinal transit are a new generation of pain treatment [50]. According to our results, 14-OMeC6SU inhibited gastrointestinal transit more pronounced than C6SU or codeine and it was similar to that of morphine. In fact, among the investigated compounds, C6SU was the least effective in this test, displaying similar gastrointestinal transit inhibition at 9- and 3-fold higher dose compared to that of 14-OMeC6SU and codeine, respectively. In other words, C6SU showed less gastrointestinal side-effect than 14-OMeC6SU, codeine and morphine in terms of opioid-induced constipation following systemic administration and no CNS symptoms—such as convulsion—which have been reported earlier for C6SU following a direct administration to CNS [17]. Nevertheless, opioid agonists of high efficacy such as 14-OMeC6SU and others with limited CNS penetration might offer stronger peripheral analgesia when we are considering different pain types, including those where the peripheral opioid receptor reserve is not altered. Actually, future studies are needed to elucidate whether or not the peripheral analgesic tolerance is developed following chronic administration of high and low efficacy compounds such as 14-OMeC6SU and C6SU, respectively in inflammatory pain. Though according to the researcher’s standpoints, high efficacy opioids are favored in terms of tolerance. In general 14-OMeC6SU produced higher agonist efficacy and stronger antinociceptive effect than C6SU. However, C6SU showed less gastrointestinal side-effect. 

## 4. Materials and Methods 

### 4.1. Animals

For mouse vas deferens (MVD) experiments male NMRI mice (35–45 g, 6–10 weeks of age) were used. Further studies were carried out on male Wistar rats weighing 140-240 g (4–7 weeks of age; tail-flick test) and 160–260 g (5–8 weeks of age; rat vas deferens (RVD), CFA and gastrointestinal charcoal meal tests). Mice and rats were obtained from Toxi-Coop Zrt. (Budapest, Hungary) and the Animal House of Semmelweis University (Budapest, Hungary), respectively. Animals were housed in the local animal house of the Department of Pharmacology and Pharmacotherapy, Semmelweis University (Budapest, Hungary).

For in vitro receptor binding assays, male Wistar rats (250–300 g body weight; 6–10 weeks of age) and male guinea pigs (~400–700 g body weight, 4–8 weeks of age; LAL/HA/BR strain) were used. Rats were purchased from and housed in the local animal house of the Biological Research Centre (Szeged, Hungary), guinea pigs were obtained from and housed in LAB-ÁLL Bt. (Budapest, Hungary).

The animals were kept in a temperature controlled room (21–24 °C) under a 12:12 light and dark cycle and were provided with water and food ad libitum. All housing and experiments were handled in accordance with the European Communities Council Directives (2010/63/EU), the Hungarian Act for the Protection of Animals in Research (XXVIII.tv. 32.§) and local animal care committee (PEI/001/276-4/2013). All efforts were made to minimize the number of animals and their suffering.

### 4.2. Chemicals

Codeine-6-*O*-sulfate (C6SU) and 14-methoxycodeine-6-*O*-sulfate (14-OMeC6SU) were synthesized as described under Section 4.3. Tris-HCl, EGTA, NaCl, MgCl_2_ x 6H_2_O, Na- HCO_3_, KCl, KH_2_PO_4_, glucose, 11.0; CaCl_2_, GDP, the GTP analog GTPγS, naloxone methiodide and the KOR agonist U-69593 were purchased from Sigma-Aldrich (Budapest, Hungary). The MOR selective agonist enkephalin analog Tyr-D-Ala-Gly-(NMe)Phe-Gly-ol (DAMGO) and the DOR selective agonist deltorphin II (Delt II) were obtained from Bachem Holding AG (Bubendorf, Switzerland). The selective DOR agonist Ile^5,6^-deltorphin II (IleDelt II) was synthesized in the Laboratory of Chemical Biology group of the Biological Research Centre of the Hungarian Academy of Sciences (Szeged, Hungary). The non-selective opioid receptor antagonist naloxone was kindly provided by the company Endo Laboratories DuPont de Nemours (Wilmington, DE, USA). Morphine and codeine hydrochloride were obtained from Alkaloida-ICN (Tiszavasvári, Hungary). Complete Freund’s Adjuvant (CFA), a water-in-oil emulsion of killed mycobacterium, was purchased from Calbiochem (San Diego, CA). For in vitro tests, all ligands were dissolved in water and were stored in 1 mM stock solution at 20 ˚C. Ligands used for in vivo assays were dissolved in saline prior to the experiments. 

The radiolabeled GTP analog, [^35^S]GTPγS (specific activity: 1250 Ci/mmol) was purchased from PerkinElmer (through Per-form kft., Budapest, Hungary). [^3^H]DAMGO (specific activity: 38.8 Ci/mmol), [^3^H]IleDelt II (specific activity: 19,6 Ci/mmol) were radiolabeled by the Laboratory of Chemical Biology group in BRC (Szeged, Hungary). [^3^H]U-69593 (specific activity: 43,6 Ci/mmol) were purchased from PerkinElmer (through Per-Form Hungária Kft., Budapest, Hungary). The UltimaGold^TM^ MV aqueous scintillation cocktail was purchased from PerkinElmer (through Per-Form Hungária Kft., Budapest, Hungary). 

### 4.3. Chemistry

#### 4.3.1. Synthesis of the Studied Compounds

The preparation of 14-methoxycodeinone was accomplished by the procedure of Kobylecki et al. [51]. 14-hydroxycodeinone was *O*-alkylated with dimethyl sulfate or methyl iodide in the presence of sodium hydride. The reduction of 14-methoxycodeinone with sodium borohydride in methanol yielded 14-methoxycodeine. It was documented that the sodium borohydride reduction of codeinone or 14-hydroxy-codeinone always proceeds streospecifically resulting in only the 6α-hydroxy derivative of codeine or 14-hydroxy-codeine but never the 6β-isomers. The hydride ion cannot attack from the α-side of the codeinone molecule because of the steric hindrance of ring E (dihydrofuran) oxygen. With the ring E is open, the reduction of 4-hydroxy-7,8-didehydro-morphinan-6-one affords the C-6 epimer secondary alcohols in equal amounts [52,53].

The preparation of 14-methoxycodeine-6-*O*-sulfate ester was accomplished by our reported method, the sulfation was performed with sulfur trioxide-pyridine complex in pyridine solvent. The structures of codeine-6-*O*-sulfate and 14-methoxycodeine-6-*O*-sulfate were elucidated by NMR spectroscopy. It is noteworthy that the influence of C-6 sulfate ester group is significant in the NMR spectrum. For example, the chemical shifts of H-1 and H-2 aromatic protons separate remarkably comparing with the chemical shifts of the parent compounds. In the case of H-5, H-6 and H-7, the resonances of the sulfate esters were deshielded, resulting in a 0.5–1.0 ppm shift compared with the corresponding values of codeine and 14-methoxycodeine. The lower field chemical shifts of the H-5, H-6 and H-7 protons are explained by the electron withdrawing effect of sulfate group [54].

#### 4.3.2. Codeine-6-*O*-sulfate

**Figure 9:**
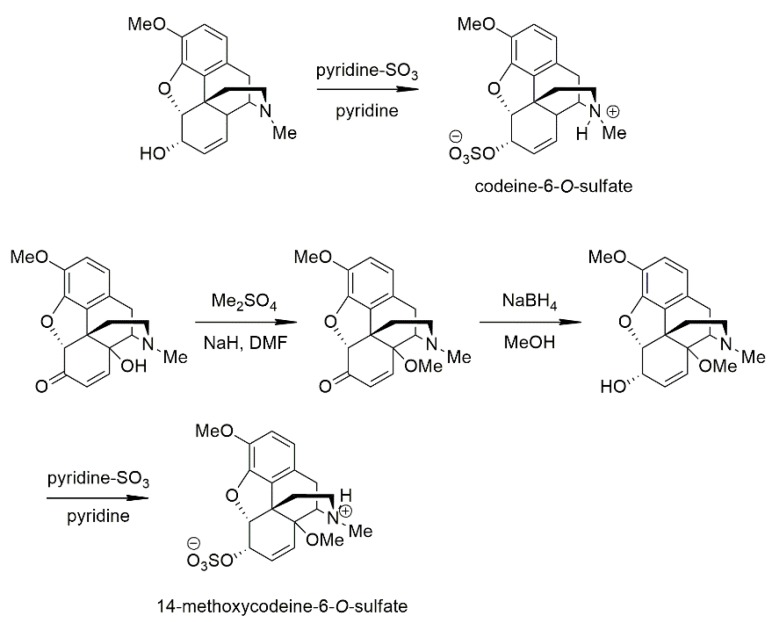
The synthesis of codeine-6-*O*-sulfate and 14-methoxycodeine-6-*O*-sulfate. For further details, see Section 4.3.

Codeine-6-*O*-sulfate was synthesized using our previously reported method [54]. Codeine (0.90 g, 3.00 mmol) was dissolved in 10 mL anhydrous pyridine. To this solution, pyridine-SO_3_ complex (1.43 g, 3 equiv.) was added in small portions and the slurry was stirred for 3.5 hours at 60 °C (Figure 9). The crude product precipitated during the reaction as a white powder. Cold water (10 mL) and chloroform (10 mL) were added to the suspension and was kept in the freezer overnight. The precipitate was collected by filtration, washed twice with cold water and crystallized from boiling water to give pure codeine-6-*O*-sulfate (colorless crystals, 37% crystallized yield). Mass spectra were recorded on Agilent 6410 Triple Quad instrument using electrospray ionization (ESI) and negative polarity. The purity of the samples was determined on HPLC system acetonitril–acetate buffer (0.02 mol, pH = 4.75, 30/70 *v*/*v*). The purity of codein-6-*O*-sulfate was > 97%. M.p.: 239–241 °C decomp. (water) C_18_H_21_NO_6_S ESI MS: 379.

^1^H-NMR (600 MHz, dmso) δ 9.68 (s, 1H, N-H), 6.74 (d, *J* = 8.2 Hz, 1H, H-2), 6.58 (d, *J* = 8.2 Hz, 1H, H-1), 5.76 (d, *J* = 9.8 Hz, 1H, H-7), 5.32–5.27 (m, 1H, H-8), 4.99 (d, *J* = 6.0 Hz, 1H, H-5), 4.62 (br d, *J* = 2.0 Hz, 1H, H-6), 4.17 (s, 1H, H-9), 3.72 (s, 3H, O-CH_3_), 3.31 (m, 2H, H-10b, H-16b), 2.92 (s, 3H, N-CH_3_), 2.86 (s, 1H, H-14), 2.86 (m, 1H, H-16a), 2.78 (m, 1H, H-10a), 2.24 (s, 1H, H-15b), 1.96 (s, 1H, H-15a). ^13^C-NMR (150 MHz, DMSO-d_6_): δ = 147.3, 141.9, 132.6, 128.9, 125.5, 123.9, 119.3, 114.0, 89.5, 70.4, 59.5, 56.0, 46.4, 41.6, 40.6, 38.2, 32.6, 20.9.

#### 4.3.3. 14-Methoxycodeine-6-*O*-sulfate

14-OH-codeinone (3.00 g, 9.10 mmol) was dissolved in anhydrous DMF (15 mL), NaH (0.75 g, 3.5 equiv.) was added in small batches and the resulting slurry was stirred for 1h at rt (Figure 9). Then it was placed on an ice bath and dimethyl sulfate (1.2 mL, ~1.4 equiv.) was added dropwise after which the mixture was stirred for 4h at rt. Water (10 mL) and 25% NH_4_OH (5 mL) was added, then solvent was evaporated under vacuum. The residue was dissolved in chloroform (100 mL), rinsed with brine, dried over anhydrous Na_2_SO_4_ (Figure 9), which was followed by the evaporation of the solvent to yield 14-*O*-methylcodeinone as a dark red oil. A portion of this crude product (2.15 g, 6.50 mmol) was dissolved in methanol (50 mL) and was placed on an ice bath while NaBH_4_ (1.5 g, ~6 equiv.) was added in small portions (Figure 9). Then, the resulting mixture was stirred for 2 h at rt. The mixture was made alkaline (pH = 9) by adding K_2_CO_3_, then the solvent was evaporated and the residue was taken up in water (50 mL) and extracted with chloroform (3 × 30 mL), rinsed with brine and dried over anhydrous Na_2_SO_4_. Removal of the solvent yielded 14-methoxycodeine as a pale yellow oil. This was dissolved in dry pyridine and esterified as described for the synthesis of codeine-6-*O*-sulfate (Figure 9). The purity of 14-methoxycodein-6-sulfate ester was > 97%. Crude 14-methoxycodeine-6-*O*-sulfate was crystallized from boiling water to yield 1.5 g pure product (colourless crystals, 59% crystallized yield). M.p. > 285 °C decomp. (water) C_19_H_23_NO_7_S ESI MS: 409

^1^H-NMR (600 MHz, DMSO-d6) δ 9.06 (s, 1H, N-H), 6.76 (d, *J* = 8.3 Hz, 1H, H-2), 6.60 (d, *J* = 8.2 Hz, 1H, H-1), 6.07 (dt, *J* = 10.0, 1.7 Hz, 1H, H-7), 5.57 (dd, *J* = 10.0, 3.3 Hz, 1H, H-8), 4.92 (dd, *J* = 6.2, 1.3 Hz, 1H, H-5), 4.86 (ddd, *J* = 5.9, 3.2, 2.1 Hz, 1H, H-6), 4.34 (d, *J* = 6.5 Hz, 1H, H-9), 3.74 (s, 3H, O-CH_3_), 3.50 (d, *J* = 19.9 Hz, 1H, H-10b), 3.20 (s, 3H, O-CH_3_), 3.19 (d, *J* = 4.6 Hz, 1H, H-16b), 2.93 (d, *J* = 4.4 Hz, 3H, N-CH_3_), 2.91–2.81 (m, 2H, H-10a, H-16a), 2.47 (td, *J* = 13.6, 4.9 Hz, 1H, H-15b), 1.86 (dd, *J* = 13.4, 3.7 Hz, 1H, H-15a). ^13^C-NMR (150 MHz, DMSO-*d*_6_): δ = 143.4, 137.4, 136.6, 131.6, 127.7, 125.1, 118.3, 115.9, 89.0, 73.7, 64.8, 56.1, 55.9, 46.4, 44.7, 42.0, 29.3, 28.6, 21.3 ppm.

### 4.4. Receptor Binding Assays

#### 4.4.1. Membrane Preparations

Animals were decapitated and their brains and spinal cords (from rats only) were quickly removed. The tissue samples were prepared for membrane preparation according to Benyhe and co-workers [55]. Membrane fractions were prepared for competition and [^35^S]GTPγS binding assays according to Zádor and co-workers [56]. Spinal cord membranes were only used for [^35^S]GTPγS binding assays. In brief, samples were homogenized and then centrifuged in ice-cold 50 mM Tris-HCl (pH 7.4) buffer and incubated at 37 °C for 30 min in a shaking water-bath. After incubation, the centrifugation was repeated and the final pellet was suspended in 50 mM Tris-HCl pH 7.4 buffer, containing 0.32 M sucrose and stored at –80 °C for further use. For the [^35^S]GTPγS binding experiments the final pellet of rat/guinea pig brain or spinal cord membrane fractions were suspended in ice-cold TEM (Tris-HCl, EGTA, MgCl_2_) buffer and stored at–80 °C for further use.

#### 4.4.2. Radioligand Competition Binding Assays

In competition binding assays the affinity of an unlabeled compound is analyzed by measuring radioligand specific binding in the presence of increasing concentrations of the unlabeled test compound [57].

Aliquots of frozen rat and guinea pig brain membrane homogenates were centrifuged (40,000 g, 20 min, 4°C) to remove sucrose and the pellets were suspended in 50 mM Tris-HCl buffer (pH 7.4). Brain membranes homogenates containing 0.3-0.5 mg/mL of protein were incubated in the presence of increasing concentrations (0.1 nM-10 µM) of C6SU, 14-OMeC6SU, codeine or with the equivalent homologues of the radioligands (DAMGO, Ile^5,6-^deltorphin II and U-69593 for control) with ~ 1-3 nM concentrations of the given radioligand. The incubation temperature and time were based on the correspondent radioligand and were the following: [^3^H]DAMGO and [^3^H]Ile^5,6-^deltorphin II in 35 °C for 45 min, [^3^H]U-69593 in 30°C for 30 min. Experiments with [^3^H]U-69593 were performed in guinea pig brain membrane homogenates, since the guinea pig brain has significantly more KORs than the rat brain, while the rest of the radioligands ([^3^H]DAMGO and [^3^H]Ile^5,6-^deltorphin II) were incubated together with rat brain membrane homogenates. Non-specific and total binding was determined in the presence of 10 μM unlabeled naloxone and in the absence of unlabeled compounds, respectively. The reaction was terminated by rapid filtration under vacuum (Brandel M24R Cell Harvester), and washed three times with 5 mL ice-cold 50 mM Tris-HCl through Whatman GF/C ([^3^H]DAMGO, [^3^H]Ile^5,6-^deltorphin II or GF/B ([^3^H]U-69593) glass fibers (GE Healthcare Life Sciences through Izinta Kft., Budapest, Hungary). The radioactivity of the filters was detected in UltimaGold^TM^ MV aqueous scintillation cocktail with Packard Tricarb 2300TR liquid scintillation counter. The competition binding assays were performed in duplicate and repeated at least three times.

#### 4.4.3. Functional [^35^S]GTPγS Binding Assays

In [^35^S]GTPγS binding experiments we measure the GDP→GTP exchange of the G_αi/o_ protein in the presence of the test compound in increasing concentrations to measure ligand potency and the maximal effect (efficacy) of receptors G-protein [58]. The nucleotide exchange is monitored by a radioactive, non-hydrolysable GTP analog, [^35^S]GTPγS.

The functional [^35^S]GTPγS binding experiments were performed as previously described [59,60], with modifications. Briefly, the rat or guinea pig brain or rat spinal cord membrane homogenates containing ~10 μg/mL protein were incubated at 30 °C for 60 min in Tris-EGTA buffer (pH 7.4) composed of 50 mM Tris-HCl, 1 mM EGTA, 3 mM MgCl_2_, 100 mM NaCl. The incubation mixture also contained 0.05 nM [^35^S]GTPγS and increasing concentrations (0.1 nM-10 µM) of C6SU, 14-OMeC6SU, codeine, morphine, DAMGO, deltorphin II or U-69593 and excess GDP (30 µM) in a final volume of 1 mL. Experiments examining KOR activity were performed only with guinea pig brain membrane homogenates. To demonstrate the low reserve of KORs in rat brain, U-69593 was measured in these samples in the same experimental set up as described above. Finally, specific binding of [^35^S]GTPγS was also measured in the combination of 10 or 100 µM of 14-OMeC6SU in rat, or guinea pig brains, respectively with 10 µM naloxone.

Total binding was measured in the absence of test compounds, while non-specific binding was determined in the presence of 10 µM unlabeled GTPγS. The bound and unbound [^35^S]GTPγS were separated as described in previous section through Whatmann GF/B glass fibers (GE Healthcare Life Sciences through Izinta Kft., Budapest, Hungary). The radioactivity of the filters was also detected as described in the previous section. [^35^S]GTPγS binding experiments were performed in triplicates and repeated at least three times. 

### 4.5. Isolated Organs

#### 4.5.1. Mouse vas Deferens (MVD)

*Vasa deferentia* were taken out from male mice. The preparation and the experimental procedures were done as described previously [61]. Briefly, *vasa deferentia* were cleaned out from tissues and suspended between two electrodes in organ baths of 5mL volume with 0.1g initial tension. The upper and the lower electrodes have ring and straight form, respectively. The organ baths were filled with Mg^2+^ free Krebs solution, of the following composition (mM/L): NaCl, 118.0; Na-HCO_3_, 25.0; KCl, 4.7; KH_2_PO_4_, 1.2; glucose, 11.0; CaCl_2_, 2.5 aerated with carbogen (95% O_2_ + 5% CO_2_) and kept at 31°C. The stimulation parameters were as follows: field stimulation, pairs (100 ms pulse distance) of rectangular impulses (1 ms pulse width, 9V/cm i.e., supramaximal intensity) were repeated by 10 s. The muscle contractions were monitored by LabChart 6.0 software.

#### 4.5.2. Rat vas Deferens (RVD)

*Vasa deferentia* were removed from Wistar male rats and the experimental procedure was as described for MVD, with the following modifications: use of Krebs solution with Mg^2+^, 0.5 g initial tension and the electrical field stimulation (pulse width,1 ms; intensity, 9 V/cm) was delivered at 0.1 Hz frequency.

#### 4.5.3. Experimental Paradigms of MVD and RVD

The experimental paradigm was similar as described previously [22]. Briefly, after the equilibration time (30-40 min and 90-120 min for MVD and RVD, respectively) the first dose of agonist was added and the concentration-effect curves were constructed in a cumulative manner. After that the preparations were washed and allowed to regain their pre-drug twitch height. Then *vasa deferentia* were equilibrated with antagonist for 20 min, and without washing a single concentration of agonist was added. In some experiments antagonists were added cumulatively followed by 20 min equilibration time. To determine dissociation constants of the antagonist, dose ratio (DR) values were obtained by the single-dose method described by Kosterlitz and Watt [62].

### 4.6. Thermal Acute Pain Model (Tail-flick Test)

The rat tail-flick test was performed in order to analyze the acute antinociceptive effect of 14-OMeC6SU and C6SU. The test compounds were dissolved in saline and administered subcutaneously (s.c.) or intracerebroventricularly (i.c.v.) as previously described [6]. Drugs or saline delivered in a volume of 2.5 mL/kg for s.c. administration (under skin over the neck), 10 µL/rat for i.c.v. injections. The experiments were carried out as described earlier [63]. Briefly, a beam of light was focused onto the dorsum of the lower third of the rat tail. Then, the time latencies until the rats flick their tails were determined before (baseline) and after injection of the test compounds. Eight seconds was used as a cut off time in order to avoid tissue damage. The antinociceptive activity was assessed 30 and 60 min after s.c. drug administration and 10, 20 and 30 min after i.c.v. administration.

### 4.7. Inflammatory Pain Model (CFA-Evoked Hyperalgesia)

For inducing inflammation, rats were injected intraplantarly (i.pl.) on the right hind paw under brief isoflurane (Sigma-Aldrich, Budapest, Hungary) anaesthesia with 150 µL CFA as described previously [5]. This treatment consistently produces localized inflammation of the inoculated paw, characterized by an increase in paw volume, paw temperature and infiltration with various types of immune cells [64]. Following the 4th and 7th day after the i.pl. CFA injection, baseline (to pretest compound) paw pressure thresholds (PPTs) of the inflamed and noninflamed paws were determined by paw pressure algesiometry (modified Randall–Selitto test; Ugo Basile, Comerio, Italy) as described in detail previously [37,65]. PPTs were then re-evaluated at 30 and 60 min after s.c. drug administration in the indicated dosages, using an arbitrary cut-off weight of twice the control and expressed in grams. Additionally, the peak effect dosage of the given test compound was blocked by the peripheral restricted naloxone methiodide to assert the peripheral opioid receptor mediation. 

### 4.8. Determination the Effect of Test Compounds on Gastrointestinal Transit

In order to determine the effect of 14-OMeC6SU and C6SU on the gastrointestinal transit and to compare to that of codeine and morphine, the charcoal meal test was applied in rats, as described before [23]. Briefly, male Wistar rats were fasted 18 h prior to the experiments, with free access to water. After the fasting period, a charcoal suspension (10% charcoal in 5% gum arabic) was given in a volume of 2 mL/animal by an oral gavage. A total of 30 min later the rats were euthanized, their entire small intestines were removed and the distance travelled by the charcoal suspension was measured and compared to the total length of small intestine. 14-OMeC6SU, C6SU, codeine and morphine were given s.c. at various doses, in a volume of 0.25 mL/100 g, 30 min before the application of the charcoal suspension and 60 min before the assessment of distance of the charcoal travel. The applied doses were based on those used in the rat tail-flick test and considering the time-lag. 

### 4.9. Data Analysis

#### 4.9.1. Receptor Binding Assays

The specific binding of the radiolabeled compound ([^3^H]ligand, [^35^S]GTPγS) was calculated by the subtraction the level of non-specific binding from the level of total binding and was given in percentage. Data were normalized to total specific binding, settled 100%, which in case of [^35^S]GTPγS also represents the level of basal activity of the G-protein. The means ± S.E.M. of data sets were plotted in the function of the applied ligand concentration range in logarithm form and were fitted with the professional curve fitting program, GraphPad Prism 5.0 (GraphPad Prism Software Inc., San Diego, CA), using non-linear regression. In the radioligand competition binding assays the ‘Dose-response - Inhibition’ equation was applied to determine IC_50_ (unlabeled ligand affinity) and to further calculate the inhibitory constant (K_i_) value according to the Cheng-Prusoff equation [66]. Selectivity ratios were calculated based on the K_i_ values. In case of [^35^S]GTPγS binding assays the ‘Dose-response - Stimulation’ equation was applied to obtain the maximum G-protein efficacy (E_max_) and ligand potency (EC_50_), respectively.

For two data sets unpaired Student’s t test with two-tailed P value for more than two data sets One-way ANOVA, with Holm-Sidak’s multiple comparison test was used. One sample t-test with a hypothetical value of 100% was applied when given specific binding values were compared to total specific binding (100%) in receptor binding assays. Statistical analysis was performed with GraphPad Prism 5.0 program; significance was accepted at *P* < 0.05 level.

#### 4.9.2. MVD and RVD Bioassays

The means ± S.E.M. of data sets were plotted in the function of the applied ligand concentration range in logarithm form and were fitted with non-linear regression in GraphPad Prism 5.0 (GraphPad Prism Software Inc., San Diego, CA), using ‘Dose-response-Stimulation’ equation. From the concentration-response curves the 50% effective concentration (EC_50_) and maximal effect (E_max_) were determined. In MVD, the equilibrium dissociation constant of naloxone (K_e_) of opioid receptor selective antagonist, naloxone (MOR), naltrindole (DOR) and nor-BNI (KOR) were also calculated in the presence of the test compounds with the single-dose method as described previously [62]. Antagonist affinities (K_e_) were calculated as follows: Ke = [antagonist concentration]/[dose ratio]-1.

#### 4.9.3. Rat Tail-flick Test, Gastrointestinal Transit and CFA-Evoked Hyperalgesia Tests

In RTF test, after the dose-response curves were constructed the dose necessary to produce a 50% effect (ED_50_) and 95% confidence limits were calculated by the Litchfield–Wilcoxon method [67]. In case of gastrointestinal transit test the distance travelled by the charcoal suspension was expressed as a percentage of total small intestine length. Significance was determined by One-way ANOVA with Tukey’s multiple comparisons test. In CFA-evoked hyperalgesia test results between and within noninflamed-inflamed group were compared with two-way ANOVA using Sidak’s or Tukey’s multiple comparisons test, respectively. Statistical analysis for the described experiments here were performed with GraphPad Prism 5.0 program, while the significance level was accepted at *P* < 0.05.

## 5. Conclusions

14-OMeC6SU proved to be a MOR agonist of higher antinociceptive potency and efficacy, than the parent compound C6SU or codeine. Systemic C6SU has an antinociceptive effect of ceiling pattern in thermal pain model. 14-OMeC6SU in certain doses showed peripheral antihyperalgesic effect in the inflammatory pain model. Despite the analgesic ceiling effect of systemic C6SU compared to 14-OMeC6SU, codeine or morphine in thermal pain model under present experimental conditions, C6SU showed peripheral antihyperalgesic effect with fewer gastrointestinal side effects. 

## Figures and Tables

**Table 1:** Inhibitory constant values (K_i_ ± S.E.M.) and selectivity ratios of codeine-6-*O*-*sulfate* (C6SU) and 14-methoxycodeine-6-*O*-sulfate (14-OMeC6SU) compared to codeine in competition binding assays with [^3^H]DAMGO, [^3^H]IleDelt II and [^3^H]U-69593, which are μ type opioid receptor (MOR), δ type opioid receptor (DOR) and κ type opioid receptor (KOR) specific radioligands, respectively performed in rat or guinea pig brain membrane homogenates. The unlabeled form of the radioligands are also indicated for control and for further comparison.

	K_i_ ± S.E.M. (nM)			Selectivity Ratio		
Compounds	[^3^H]DAMGO (μ) ^1^	[^3^H]IleDelt II (δ) ^1^	[^3^H]U-69593 (κ) ^2^	δ/μ	κ/μ	δ/κ
**14-OMeC6SU**	3.37 ± 0.48 ** (n = 6)	345.52 ± 132.52 (n = 5)	245.57 ± 215.01 (n = 5)	102.5	72.9	1.4
**C6SU**	96.91 ± 20.3 * (n = 5)	968.28 ± 471.38 (n = 6)	N.D.^3^ (n = 5)	9.9	-	-
**Codeine**	736.74 ± 319.63 (n = 4)	N.D. ^4^ (n = 4)	N.D. ^4^ (n = 4)	-	-	-
**Morphine ^5^**	0.76 ± 0.08 (n = 7)	114.21 ± 44.83 (n = 6)	N.D. ^4^ (n = 4)	150.3	-	-
**Homologous ligand^6^**	0.59 ± 0.12 (n = 5)	3.81 ± 0.88 (n = 6)	5.51 ± 0.97 (n = 5)	-	-	-

^1^ performed in rat brain membrane homogenates; ^2^ performed in guinea pig brain membrane homogenates; ^3^ the compound did not inhibit total specific radioligand binding (100%) to 50%, thus the K_i_ value cannot be interpreted (N.D. not determined); ^4^ the compound did not alter significantly (One-sample t test) the total specific radioligand binding (100%), thus the K_i_ value cannot be interpreted (N.D. not determined); ^5^ adopted from [23]; ^6^ indicates the unlabeled form of the radioligands and represent a control for the assay (μ: DAMGO δ: IleDelt II, κ: U-69593); * compared to codeine (One-way ANOVA, with Sidak’s multiple comparison test; ** *P* < 0.01, *** *P* < 0.001).

**Table 2:** Maximum G-protein efficacy (E_max_) and potency (EC_50_) of 14-methoxycodeine-6-*O*-sulfate (14-OMeC6SU) compared to codeine-6-*O*-sulfate (C6SU) and codeine in [^35^S]GTPγS binding assays in rat or guinea pig brain and rat spinal cord membrane homogenates. The table also indicates the µ-, δ- and κ-opioid receptor specific agonists DAMGO, deltorphin II (Delt II) and U-69593, respectively.

Compounds	Tissue Samples (*n*)	E_max_ ± S.E.M. (%)	EC_50_ ± S.E.M. (nM)
**14-OMeC6SU**	Rat brain (7)	128.6 ± 3.69	301.1 ± 196.6
	Guinea pig brain (6)	130.2 ± 2.72	>1000
	Rat spinal cord (5)	128.8 ± 1.51	674.2 ± 157.9
**C6SU**	Rat brain (5)	121 ± 7.27	>10000
	Guinea pig brain (5)	101.7 ± 1.97	N.D.^1^
	Rat spinal cord (5)	119.3 ± 1.81	>1000
**Codeine**	Rat brain (5)	109.7 ± 11.48	N.D.^1^
	Guinea pig brain (5)	103.6 ± 2.04	N.D.^1^
	Rat spinal cord (4)	102.1 ± 0.49	N.D.^1^
**Morphine**	Rat brain (7) ^2^	128.8 ± 2.66	250 ± 131.7
	Guinea pig brain (5) ^3^	119 ± 1.99	461.6 ± 250.1
	Rat spinal cord (7) ^2^	124.4 ± 2.09	126 ± 72.37
**DAMGO (μ)**	Rat brain (7)	155.3 ± 4.98	427 ± 201.9
	Rat spinal cord (5)	137.8 ± 2.65	90.14 ± 40.61
**Delt II (δ)**	Rat brain (7)	122.9 ± 1.77	44.39 ± 23.51
	Rat spinal cord (3)	113 ± 3.32	>1000
**U-69593 (κ)**	Rat brain (4)	103 ± 0.84	N.D.^1^
	Guinea pig brain (7)	126.8 ± 3.05	298.3 ± 176.2

^1^ not determined, the compound did not alter significantly the basal activity (100%) of the G-protein, thus the EC_50_ value cannot be interpreted (One sample t test, hypothetical value 100%; ^2^ adopted from [35]; ^3^ adopted from [23]; * compared to data obtained from guinea pig brain membranes (One-way ANOVA, with Sidak’s multiple comparison test, *P* < 0.05).

**Table 3:** Examining the opioid receptor mediation in G-protein activity ([^35^S]GTPγS specific binding normalized to basal activity) of 14-OMeC6SU in the presence or absence of 10 µM naloxone in [^35^S]GTPγS binding assays performed in rat and guinea pig brain membrane homogenates. 14-OMeC6SU was added in 10 and 100 µM in rat and guinea pig brain membranes, respectively.

	[^35^S]GTPγS Specific Binding ± S.E.M. (%)	
Compounds	Rat Brain	Guinea Pig Brain
**14-OMeC6SU**	130.1 ± 7.99 (n = 7)	128.7 ± 2.08 (n = 5)
**+10 µM naloxone**	96.24 ± 4.51 ** (n = 5)	95.84 ± 1.34 *** (n = 5)

* compared to C6SU (unpaired t test, two-tailed *P*-value, **: *P* < 0.01; ***P < 0.001).

**Table 4:** The agonist activity of 14-OMeC6SU described by maximum efficacy (E_max_) and ligand potency (EC_50_) to inhibit electrically evoked mouse vas deferens contractions and rat vas deferens contractions (MVD and RVD, respectively). Results were compared to C6SU, codeine or to prototypic opioid agonists, morphine and DAMGO.

	E_max_ ± S.E.M. (%)		EC_50_ ± S.E.M. (nM)	
Compounds (n)	MVD	RVD	MVD	RVD
14-OMeC6SU (13; 6)	98.31 ± 0.52 ***^/###/+++^	74.81 ± 2.74 ***^/×××/$$$^	239.2 ± 6.15 ***^/###^	>1000
C6SU (10; 5)	77.81 ± 3.11 ^###/+++^	16.15 ± 3.25 ^×××^	>1000 ^##/+++/×××^	>10000
Codeine (5; 4)	40.33 ± 4.69	No effect	>1000	N.D. ^2^
Morphine (8; 6) ^1^	46.91 ± 3.23	No effect	154.5 ± 93.36	N.D. ^2^
DAMGO (8; 5)	91.91 ± 2.97	97.57 ± 4.52	122.4 ± 19.55	79.81 ± 19.61

* compared to C6SU (One-way ANOVA, with Sidak’s multiple comparison test, *** *P* < 0.001); ^#^ compared to codeine (One-way ANOVA, with Sidak’s multiple comparison test, ^###^ *P* < 0.001; ^##^ *P* < 0.01); ^+^ compared to morphine (One-way ANOVA, with Sidak’s multiple comparison test, ^+++^ *P* < 0.001); ^×^ compared to DAMGO (One-way ANOVA, with Sidak’s multiple comparison test, ^×××^ *P* < 0.001; ^××^ *P* < 0.01); ^$^ compared to 14-OMeC6SU in MVD (unpaired t-test, two tailed *P* value; ^$$$^ *P* < 0.001); ^1^ adopted from [23]; ^2^ not determined, since the compounds did not show inhibitory effect.

**Table 5:** The opioid receptor selectivity of 14-OMeC6SU in electrically evoked contractions of MVD and RVD bioassays compared to C6SU, indicated by the K_e_ value of selective opioid antagonists. Reference opioid agonists were also measured for control.

	K_e_ ± S.E.M. (nM)			
	Naloxone (µ)		Naltrindole (δ)	nor-BNI (κ)
Compounds	MVD	RVD	MVD	MVD
**14-OMeC6SU**	2.08 ± 0.16 (n = 11)	2.59 ± 0.53 (n = 4)	4.01 ± 1.06 (n = 4)	5.31 ± 1.09 (n = 4)
**C6SU**	3.25 ± 0.77 (n = 10)	N.D.^1^	2.69 ± 0.93 (n = 4)	3.05 ± 0.89 (n = 6)
**DAMGO** (µ)	1.8 ± 0.32 (n = 5)	1.87 ± 0.4 (n = 5)	N.D.^1^	N.D. ^1^
**DPDPE** (δ)	N.D.^1^	N.D.^1^	0.63 ± 0.33 (n = 6)	N.D. ^1^
**U-69593** (κ)	N.D.^1^	N.D.^1^	N.D.^1^	0.33 ± 0.14 (n = 3)

^1^ not determined.

**Table 6:** Antinociceptive potencies (ED_50_) of 14-OMeC6SU and codeine against radiant heat induced nociception in rat tail-flick test after 30 and 60 min of s.c. administration. As a reference compound morphine was also indicated.

	ED_50_ (95% Confidence Limit) (µmol/kg)	
	Time After s.c. Administration (min)	
Compounds	30	60
**14-OMeC6SU**	5.34 ^a^ (3.14–9.06)	9.88 (7.47–13.06)
**Codeine**	54.01 ^a^ (33.97–85.88)	89.86 (61.54–131.2)
**Morphine ^1^**	6.87 ^a^ (4.59–10.27)	14.17 (10.24–19.62)

^1^ [23]; ^a^ Peak of effect.

**Table 7:** Antinociceptive potencies (ED_50_) of 14-OMeC6SU against radiant heat induced nociception in rat tail-flick test after 10, 20 and 30 min of i.c.v. administration. As a reference compound morphine was also indicated. The calculated s.c./i.c.v. ratio of ED_50_ values from Table 6 are also indicated.

	ED_50_ (95% Confidence Limit) (µmol/animal)			
	Time after i.c.v. Administration (min)			ED_50_ s.c./i.c.v. Ratio
Compounds	10	20	30	
**14-OMeC6SU**	0.017 ^a^ (0.011–0.026)	0.018 (0.012–0.028)	-	314.12
**Morphine**	-	0.055^2^ (0.032–0.095)	0.039 ^a,2^ (0.022–0.068)	176.15

^2^ [22]; ^a^ Peak of effect.
